# JMJD2A participates in cytoskeletal remodeling to regulate castration-resistant prostate cancer docetaxel resistance

**DOI:** 10.1186/s12885-023-10915-1

**Published:** 2023-05-10

**Authors:** Xiang Cai, Xi Duan, Tielong Tang, Shu Cui, Tao Wu

**Affiliations:** 1 Department of Urology, Affiliated Hospital of North Sichuan Medical College, No. 1 Maoyuan South Road, Sichuan 637000 Nanchong, China; 2 Department of Dermatovenereology, Affiliated Hospital of North Sichuan Medical College, No. 1 Maoyuan South Road, Sichuan 637000 Nanchong, China

**Keywords:** Castration-resistant prostate cancer, Docetaxel resistance, JMJD2A, miR-34a/STMN1/β3-Tubulin, PDX animal model

## Abstract

**Background:**

To investigate underlying mechanism of JMJD2A in regulating cytoskeleton remodeling in castration-resistant prostate cancer (CRPC) resistant to docetaxel.

**Methods:**

Tissue samples from CRPC patients were collected, and the expression of JMJD2A, miR-34a and cytoskeleton remodeling-related proteins were evaluated by qPCR, western blot and immunohistochemistry, and pathological changes were observed by H&E staining. Further, JMJD2A, STMN1 and TUBB3 were knocked down using shRNA in CRPC cell lines, and cell viability, apoptosis and western blot assays were performed. The interaction between miR-34a/STMN1/β3-Tubulin was analyzed with dual-luciferase reporter and co-immunoprecipitation assays.

**Results:**

In clinical experiment, the CRPC-resistant group showed higher expression of JMJD2A, STMN1, α-Tubulin, β-Tubulin and F-actin, and lower expression of miR-34a and β3-Tubulin compared to the sensitive group. In vitro experiments showed that JMJD2A could regulate cytoskeletal remodeling through the miR-34a/STMN1/β3-Tubulin axis. The expression of miR-34a was elevated after knocking down JMJD2A, and miR-34a targeted STMN1. The overexpression of miR-34a was associated with a decreased expression of STMN1 and elevated expression of β3-Tubulin, which led to the disruption of the microtubule network, decreased cancer cell proliferation, cell cycle arrest in the G0/G1 phase, and increased apoptosis.

**Conclusion:**

JMJD2A promoted docetaxel resistance in prostate cancer cells by regulating cytoskeleton remodeling through the miR-34a/STMN1/β3-Tubulin axis.

**Supplementary Information:**

The online version contains supplementary material available at 10.1186/s12885-023-10915-1.

## Introduction

Prostate cancer is one of the most common malignant tumors of the male reproductive system worldwide, with a rising annual incidence rate in China. Castration-resistant prostate cancer (CRPC), often characterized by poor response to clinical treatments and short overall survival, is a type of prostate cancer that often progresses after androgen deprivation therapy (ADT). Almost all advanced prostate cancer will inevitably develop into CRPC [1]. Germline or somatic aberrations in the DNA damage repair genes are found in 19% of primary prostate cancer and almost 23% of metastatic CRPC and compromise genomic integrity. Several PARP inhibitors have been investigated in metastatic CRPC patients, however, it is regrettable that these PARP inhibitors have diverse side effects [2]. Docetaxel is a chemotherapeutic drug that has been used to target the cytoskeleton in the first-line treatment of CRPC and has shown to possess a transient palliative effect [3]. However, due to the development of docetaxel resistance after long-term use, it is associated with a low 5-year survival rate [4, 5]. Docetaxel resistance greatly limits the clinical efficacy of CRPC and is a pressing challenge in the clinical treatment of CRPC. Due to the increasing incidence of prostate cancer in China, there is an urgent need to investigate the underlying mechanisms of docetaxel resistance in CRPC.

The cytoskeletal system is mainly composed of microtubules, microfilaments and intermediate fibers, which participate in cell morphology maintenance, intracellular material transport and organelle movement. Previous studies have shown that cytoskeletal remodeling plays an important role in tumor resistance [6]. For instance, docetaxel was shown to induce microtubulin polymerization in prostate cancer xenografts, which was reduced or absent in xenograft models of CRPC patients [7]. Cytoskeletal remodeling may inhibit tumor resistance by promoting the restoration of myosin II activity [6]. At present, the influence and mechanism of cytoskeletal remodeling on docetaxel-resistant patients remain unclear.

Jumonji domain 2A (JMJD2A), a histone demethylase, was found to be upregulated in prostate cancer and indicated that it might promote prostate cancer development [8, 9]. Nakagawa et al. found that the downregulation of JMJD2A expression in gastric cancer cells significantly increased the sensitivity of the cancer cells to docetaxel [10]. However, the role and mechanism of JMJD2A expression in docetaxel-resistant CRPC have not yet been reported.

It was reported that reducing the JMJD2A promoter activity could inhibit miR-34a expression [11]. miR-34a is a short-stranded non-coding RNA that participates in regulating cell survival, migration, and remodeling, and is directly related to the G1/S cell cycle checkpoint regulatory pathway [12–14]. Studies have revealed that miR-34a contributes to the angiogenesis in prostate cancer, which may regulate endothelial cells via non-cell-autonomous, as well as cell-autonomous techniques, and therefore control angiogenesis [15]. Additionally, miR-34a can inhibit the proliferation, migration, invasion and tumor formation of prostate cancer cells [16] and reduce docetaxel resistance in prostate cancer patients [17]. Stathmin 1 (STMN1) is a highly conserved intracellular protein that can bind to α/β microtubulin to depolymerize microtubules. Studies have shown that STMN1 is highly expressed in various cancers, including gastric, ovarian, and prostate cancers [18–20], and its increased expression was associated with resistance of tumor cells to the anti-tumor effects of microtubule inhibitors. Vetter et al. found that the miR-34a/STMN1/β3-Tubulin axis could regulate the microtubule network of tumor cells in osteosarcoma, whereby miR-34a directly inhibited STMN1 expression and upregulated β3-Tubulin, leading to microtubule network disruption and cancer cell death [20]. Further, it was reported that high STMN1 expression was positively correlated with docetaxel resistance [21]. Therefore, we speculated that JMJD2A might regulate the cytoskeleton remodeling of CRPC docetaxel resistance through the miR-34a/STMN1/β3-Tubulin axis.

In this study, we combined clinical sample studies and designed in vivo and in vitro experiments to explore the significance and mechanism of JMJD2A and cytoskeleton remodeling in docetaxel-resistant CRPC and identify an effective therapeutic approach for overcoming docetaxel-resistant CRPC.

## Materials and methods

### Clinical sample collection

Cancer and paracancerous tissue samples of CRPC docetaxel-resistant patients and CRPC docetaxel-sensitive patients were collected during radical prostatectomy or prostate biopsy. The clinical data of the patients, including pathology, diagnosis, PSA level, Gleason score, clinical stage, etc., were also retrieved. The study was performed following the principles of the Declaration of Helsinki, and the patients or their family members provided signed informed consent. All experimental procedures were approved by the Medical Ethics Committee of the Affiliated Hospital of North Sichuan Medical College (2022ER246-1).

### Animals

Sixteen male NSG mice (20–30 g) aged 6 to 7 weeks were purchased from Sipeifu Biotechnology Co., Ltd. (Beijing, China). The animals were kept in a specific pathogen-free (SPF) animal room with a 12 h light/dark cycle, 22 ± 2 °C room temperature, 50-60% humidity and free access to food and drinking water. All the experiments were approved by the Laboratory Animal Ethics Committee of Zhejiang Haikang Biological Products Co., Ltd (HKSYDWLL2021008) and the National Research Council’s Guide for the Care and Use of Laboratory Animals.

### Cell culture

The human prostate cancer cell lines PC3 and DU145 were purchased from Guangzhou RiboBio Co., Ltd. (Guangzhou, China). The cells were cultured in RPMI-1640 medium (Hyclone) supplemented with 10% fetal bovine serum (FBS), 100 mg/mL streptomycin and 100 U/mL penicillin in a humidified incubator at 37 °C with 5% CO2. The culture medium was changed every 3 days, and the cells were collected for experiments when they reached the logarithmic growth phase.

### Establishment of drug-resistant cell lines

Docetaxel-resistant strains were derived from the parental PC3 and DU145 cell lines, as previously reported [22, 23]. Briefly, the docetaxel-resistant strains were constructed using a concentration gradient method at an initial concentration of 4 nM. After 48 h of culture, the medium was changed to a conventional medium, and the cells were allowed to recover for 2 to 3 weeks. Based on the above, the drug concentration was gradually increased until the cells could grow stably in the medium at a concentration of 100 nM and passaged to obtain docetaxel-resistant strains.

### Reagents and antibodies

Antibodies against JMJD2A, STMN1, β3-Tubulin, α-Tubulin and β-Tubulin were purchased from Cell Signaling Technology (Boston, USA). F-actin and PSA were purchased from Thermo Fisher (Shanghai, China), TUNEL Apoptosis Assay Kit from Beyotime (Shanghai, China), Phosphate-buffered saline (PBS) from Solarbio (Beijing, China), and Cell Counting Kit-8 from BBI Life Sciences (Shanghai, China).

### qRT-PCR assay

qRT-PCR assay was performed to detect the expression of miR-34a in cells. Total RNA was isolated from prostate cancer cells and prostate cancer tissue samples using the Trizol reagent (Invitrogen). First-strand cDNA was synthesized using the TransScript RT/RI and gDNA Remover (AT341, TransGen, Beijing, China). The primer sequences of hsa-miR-34a-3p were: forward, 5’-ACACTCCAGCTGGGCAATCAGCAAGTAAT-3’ and reverse, 5’-CTCAACTGGTGTCGTGGAGTCGGCAATTCAGTTGAGTGACGGGA-3’, and those of U6 were: forward, 5’-ACATGGTTGGCCTTGAGAAC-3’ and reverse, 5’-AGGGTGTTGGCTGTACTTGC-3’.

### Lentivirus Packaging

sh-JMJD2A, oe-JMJD2A and corresponding shNC control lentivirus particles were purchased from GenePharma. The sequences of JMJD2A were: 5’-CCGGAATTCATGGCTTCTGAATCAGAAAC-3’ (forward) and 5’-CGCGGATCCCTACTCCATGATGGCCCGGT-3’ (reverse). The shRNA sequence of shSTMN1 was 5’-ACCCACAAAATGGAAGCTAATAA-3’, and that of shTUBB3 was 5’-TGCACTGGTACACGGGCGAGGGC-3’. Lentivirus expressing human JMJD2A was generated by sub-cloning human JMJD2A cDNA (NM_014663.2) to the pSLIK lentivirus expression system. For lentiviral packaging, HEK293T cells were co-transfected with the lentiviral particles. For transduction, the cells were incubated with the virus-containing supernatant in the presence of 5 µg/ml polybrene. After 48 h, the infected cells were incubated for 72 h with puromycin (2 µg/ml). Then, the cells of sh-JMJD2A and oe-JMJD2A were constructed and categorized into the following groups: control oeNC, oeJMJD2A, shNC and shJMJD2A. On the basis of shJMJD2A and shNC cells, miR-34a inhibitors or NC inhibitors were added and divided into the following 4 groups: (1) shNC + NC inhibitors group, (2) shNC + miR-34a inhibitors, (3) shJMJD2A + NC inhibitors, and (4) shJMJD2A + miR-34a inhibitors. On the basis of NC inhibitors and miR-34a inhibitors cells, shSTMN1 and shNC cells were constructed and divided into the following 4 groups: (1) NC inhibitors + shNC group, (2) NC inhibitors + shSTMN1, (3) miR-34a inhibitors + shNC, and (4) miR-34a inhibitors + shSTMN1. Lastly, on the basis of shSTMN1 and shNC cells, shTUBB3 and shNC cells were constructed and divided into the following 4 groups: (1) shNC group, (2) shNC + shTUBB3, (3) shNC + shSTMN1, and (4) shSTMN1 + shTUBB3.

### Western blot

The tumor tissues and cells were lysed using the RIPA lysis buffer containing PMSF and a phosphatase inhibitor (CWbiotech, Beijing, China). They were centrifuged at 12,000×g and 4 °C for 5 min to collect the total cell lysates. Afterward, the protein content in the supernatants was determined using the BCA Protein Assay Kit (Solarbio, Beijing, China). Then, 20 µg of the corresponding protein extracts were used, separated on 10% SDS-PAGE gels, and transferred onto a PVDF membrane (GE Healthcare Life). After washing with tris-buffered saline (TBST), the membranes were blocked with 5% fat-free milk for 1 h at room temperature and incubated overnight at 4 °C with primary antibodies against JMJD2A (1:1000), STMN1 (1:1000) and β3-Tubulin (1:1000). On the following day, the membranes were washed three times for 10 min each with TBST, then incubated at room temperature for 2 h with goat and anti-rabbit IgG secondary antibodies (Cell Signaling Technology, Boston, USA). Immunoreactive proteins were visualized using the ECL chemiluminescence kit (Solarbio, Beijing, China). GAPDH was used as the internal reference. Band intensities were determined using the Chemi Capture (CLiNX, Shanghai, China) software.

### Hematoxylin-eosin (H&E) staining

H&E staining was performed to evaluate the pathological features of the clinical tissues or tumors, which were first subjected to paraffin embedding, cut into 4 μm-sections, then underwent routine H&E staining. The images were captured under a microscope.

### Apoptosis assay

Apoptosis was detected using the FITC Annexin V Cell Apoptosis Detection Kit (BD Pharmingen). Briefly, the cells were seeded into 6-well plates and cultured for 24 h. After washing twice with cold PBS, the cells were resuspended in 100 µl of 1× binding buffer at a density of 1 × 106 cells/ml and incubated with 5 µl of FITC Annexin V and propidium iodide (PI) for 15 min at room temperature away from light. Then, the cells were diluted with 400 µl of 1× binding buffer, and the apoptotic rates (including early and late apoptosis) were measured with a flow cytometer (CytoFlex S, Beckman Coulter, CA, USA).

### Cell cycle analysis

The cell cycle distribution was assessed by flow cytometry. Cells were fixed in 75% ethanol and centrifuged at 5,000 rpm at room temperature for 2 min. RNase A was added and the cells were incubated at room temperature for 30 min before adding propidium iodide (PI) and incubating at room temperature for 1 h. Subsequently, after adding 300 µL of PBS to each sample, the stained cells were analyzed on a flow cytometer using FlowJo software (BD Biosciences). The percentages of cells in G0/G1, S, and G2/M phases were shown.

### TUNEL staining

Cancer tissues were fixed in formalin, dehydrated, embedded in paraffin and sliced into 3–5 μm. TUNEL staining was performed using the One-Step TUNEL Apoptosis Assay Kit (Beyotime), following the manufacturer’s instructions. Then, the samples were incubated with TUNEL solution for 2 h at 37 °C, counterstained with PI, and observed under a fluorescence microscope (Axioskop 2; Zeiss) at 200× magnification.

### Cell viability assay

Cell viability was evaluated using the Cell Counting Kit-8 (CCK-8) assay. The cells were seeded into 96-well plates at a density of 5000 cells/well and cultured for 24 h. Afterward, 10 µl of CCK-8 solution (BBI Life Sciences) was added to each well and incubated for 1 h at 37 °C in the dark. Absorbance at 450 nm was measured using a microplate reader (Multiskan MK3, Thermo Labsystems).

### Enzyme-linked immunosorbent assay (ELISA)

The levels of serum PSA were determined using an ELISA kit (Solarbio, Beijing, China) following the manufacturer’s instructions. Absorbance was measured at 450 nm with a microplate reader.

### Xenograft Experiment

Patient-derived xenograft (PDX) models were established as previously described [24]. Briefly, the collected patient’s tumor tissues were cut into 1–3 mm3 tumor fragments and implanted subcutaneously in the lateral abdomen of the NSG mice under general anesthesia. The tumor growth was monitored by measuring the length and width of the tumor with vernier calipers, and the volume was calculated. Tumor samples from the PDX model were collected when the xenografts had grown to 1–2 cm3. They were then cut into small fragments and transplanted into other mice. Subsequent experiments were performed using PDX from the third generation onwards after initial implantation. The PDX animal models were divided into control group (CRPC docetaxel-sensitive) and resistant group (CRPC docetaxel-resistant) according to the difference of implanted clinical samples. The tumor size was measured with a vernier caliper every three days, and the tumor volume was calculated (tumor volume calculation formula: volume = 0.5×length×width^2^). When the tumor volume of the PDX model mice was about 80–100 mm^3^, the bilateral testes were surgically removed by castration, and docetaxel (2 mg/kg, twice a week) was administered by tail vein injection. After 30 days, the peripheral blood of the experimental mice was collected and euthanized with high-dose pentobarbital sodium. Tumor samples from the PDX model were collected and frozen or fixed in 4% paraformaldehyde. The animals were kept in captivity under appropriate SPF conditions.

### Co-immunoprecipitation (Co-IP) assay

Co-IP assay was performed at a temperature of 4 °C. Briefly, PC3 and DU145 cells were washed with PBS and incubated with RIPA lysis buffer for 30 min on ice. After centrifugation at 20,000×g for 10 min at 4 °C, the supernatant was collected and incubated with STMN1 antibody or control IgG for 2 h at 4 °C, followed by overnight incubation with 20 µl of Protein A/G PLUS-Agarose beads (Bimake) at 4 °C. Next, the beads were washed 4 times with RIPA, resuspended in 1×loading buffer and boiled for 5 min. Then, the supernatant was collected, separated on SDS-PAGE and transferred onto a PVDF membrane. After blocking with TBST containing 5% non-fat milk for 1 h at room temperature, the membrane was incubated overnight at 4 °C with primary antibodies against β3-Tubulin (Cell Signaling Technology). The following day, the membrane was washed 3 times with TBST and incubated with indicated secondary antibodies (Cell Signaling Technology) for 1 h at room temperature. Target proteins were detected using an ECL detection system (Tanon, Shanghai, China).

### Dual-luciferase reporter assay

The 3’-UTR of human STMN1 was amplified by PCR and cloned into the Pmir-REPORT™ miRNA expression reporter vector (Ambion, TX, USA) to obtain the STMN1 3’-UTR wild-type (WT) firefly luciferase reporter gene. Overlap PCR was performed, and mutations were introduced into the seed sequences of all four predicted miR-34a target sites within the STMN1 3’-UTR and the STMN1 3’-UTR mutant (MT) was generated. Similarly, the STMN1 3’-UTR MT was digested and ligated to the multi-cloning sites of the Pmir-REPORT miRNA expression reporter plasmid. All the recombinant DNAs were verified by DNA sequencing. HEK293T cells were inoculated onto 24-well plates and co-transfected with luciferase reporter constructs containing wild-type or mutant STMN1 3’-UTR firefly luciferase reporters and miR-34a mimics or miR-NC. Luciferase activities were detected 48 h after the transfection using the dual-luciferase Reporter Assay System (Promega, WI, USA). Firefly luciferase activity was normalized to the renilla luciferase activity.

### Immunohistochemical (IHC) detection

IHC samples were obtained from the CRPC patients’ tumor tissue and xenograft tumors from mice. The tumor specimens were fixed with 4% formalin for 48 h and embedded in dehydrated paraffin. The embedded slices were cut into 5 μm. The sections were then dewaxed, rehydrated, and heated under high pressure in citrate buffer for 15 min to restore antigen activity. Next, they were incubated with 3% hydrogen peroxide in methanol for 10 min to eliminate endogenous peroxidase activity. After blocking, the sections were incubated with α-Tubulin, β-Tubulin or F-actin-specific rabbit polyclonal antibody at 4 °C overnight. Then, the sections were washed with PBS and incubated with a secondary antibody for 20 min at room temperature. The sections were stained with DAB staining solution, counterstained with hematoxylin and detected under a microscope (Olympus CX41, Olympus company, Japan).

### Statistical analysis

All statistical analyses were performed using the SPSS 22.0 or GraphPad Prism 8.0 software. Categorical variables were expressed as numbers or percentages, and continuous variables were expressed as mean ± standard deviation. Categorical variables were analyzed using Fisher’s exact test, and the t-test was used for comparison between groups to analyze continuous variables. The chi-square test was used to compare the expression of JMJD2A, miR-34a, STMN1 and β3-Tubulin in the tissue samples of the CRPC docetaxel sensitive and drug-resistant groups. The Spearman rank test was used to analyze the correlation between the above molecules’ expression and docetaxel resistance in CRPC. *P* < 0.05 was used for determining statistical significance.

## Results

### Correlation of JMJD2A expression, Cytoskeletal Remodeling and Docetaxel Resistance in CRPC Patients

**Fig. 1:**
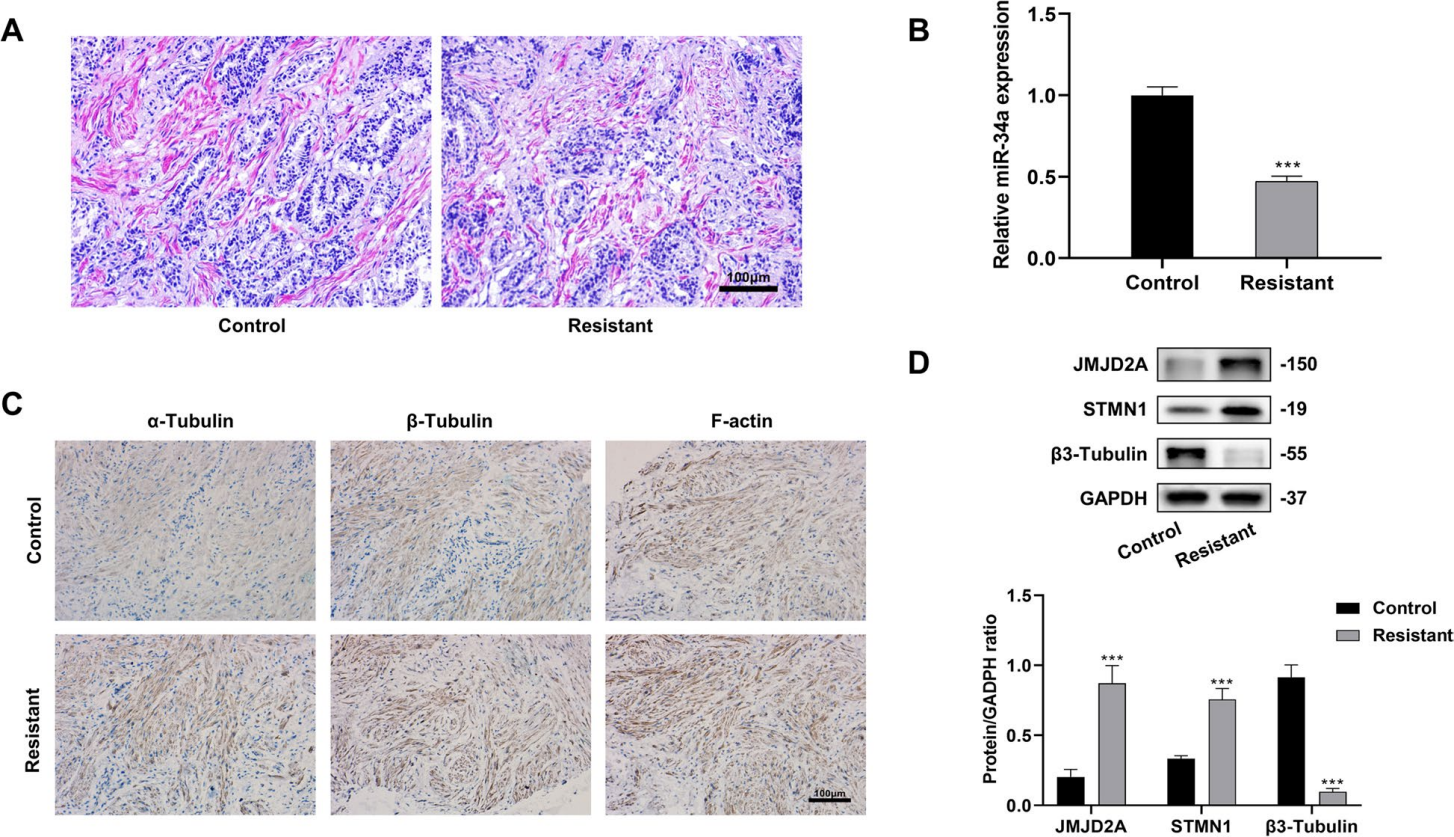
In vitro assessment of JMJD2A and cytoskeleton remodeling-related factors in CRPC docetaxel-resistant patients. **A** H&E staining to observe the pathological changes in clinical tissue samples; **B** PCR to detect the expression of miR-34a; **C** Immunohistochemistry to observe the content of microtubulin α-Tubulin and β-Tubulin, F-actin in clinical tissue samples; **D** Western Blot to detect the protein expression of JMJD2A, STMN1 and β3-Tubulin. ***P < 0.001, expression compared with the control group

The CRPC patients were divided into control group (CRPC docetaxel-sensitive) and resistant group (CRPC docetaxel-resistant) based on their response to docetaxel. Then, their cancer tissue samples were collected for H&E staining. The results showed that the prostate tissues of the control group had obvious infiltration of neoplastic glands, visible glandular cavity and were surrounded by stroma. In contrast, the tissues of the resistant group showed deeper integration, cribriform shape, and more severe damage in their glandular structure (Fig. 1A). qRT-PCR analysis showed that the expression of miR-34a in the resistant group was significantly lower than in the control group (*P* < 0.001) (Fig. 1B). Immunohistochemical results showed that the expressions of cytoskeleton-related proteins α-Tubulin, β-Tubulin and F-actin in the resistant group were significantly increased compared with the control group, indicating that docetaxel resistance was associated with cytoskeleton proliferation (Fig. 1C). In addition, compared with the control group, western blot analysis identified higher protein expression of JMJD2A and STMN1 in the resistant group but comparatively lower expression of β3-Tubulin (Fig. 1D, *P* < 0.001).

### Role of JMJD2A expression and cytoskeletal remodeling in Docetaxel-Resistant CRPC in vivo

**Fig. 2:**
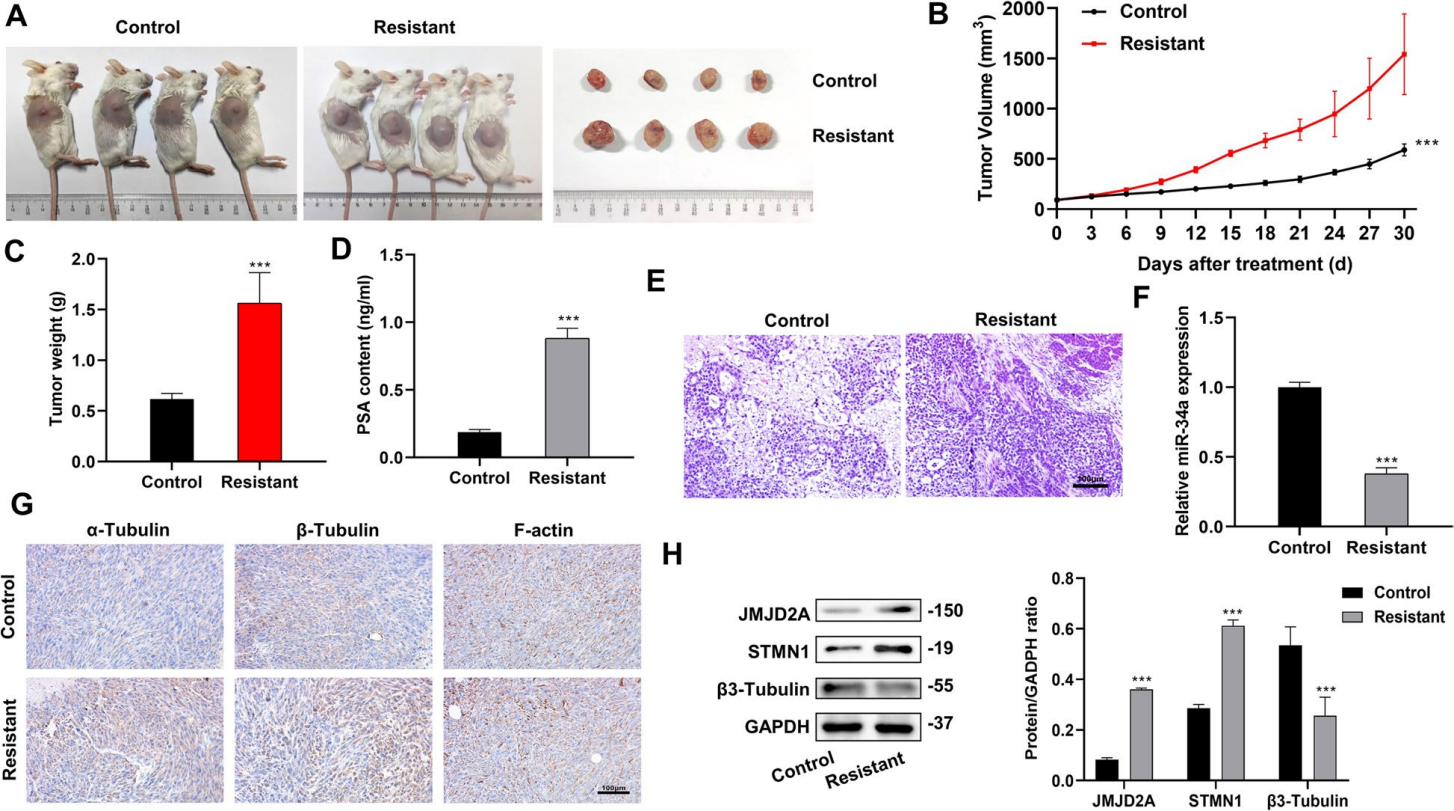
In vivo experiments validating the expression of JMJD2A and cytoskeleton remodeling-related factors in CRPC docetaxel-resistant mice. **A** Mice PDX model of the control and resistant groups; **B** Change in the tumor volume of the PDX models after treatment; **C** Change in the tumor weight of the PDX models after treatment. **D** PSA levels in mouse serum detected by ELISA; **E** The pathological changes in tissue samples observed by H&E staining; **F** miR-34a expression detected by PCR; **G** The levels of microtubulin α-Tubulin and β-Tubulin, F-actin in tissue samples observed by immunohistochemistry; **H** JMJD2A, STMN1 and β3-Tubulin protein expression detected by western blot. ***P < 0.001, compared with the control group

To further validate the relationship between JMJD2A expression and cytoskeleton remodeling in the docetaxel-resistant CRPC, PDX animal models were established using the above clinical samples. Both groups of mice were castrated and treated with docetaxel. The tumor size of the mice was measured, and their volume was calculated. Our results showed that the treatment effect of docetaxel on mice from the resistant group was poor, and their tumor volume and weight were significantly greater than the control group (Fig. 2A-C, *P* < 0.001). ELISA results showed that the serum PSA level of mice from the resistant group was significantly higher than the control group (Fig. 2D, *P* < 0.001). H&E staining was performed after castration and docetaxel treatment to observe the changes in histological morphology and evaluate the corresponding therapeutic effects. The results showed a decrease in tumor cell numbers and an increase in the matrix components in the sensitive group, while no significant alteration was observed in the architecture of the tumor tissues in the resistant group (Fig. 2E). Consistent with the results from clinical tissue samples, compared with the control group, the resistant group demonstrated lower relative mRNA expression of miR-34a and β3-Tubulin and higher expression of α-Tubulin, β-Tubulin, F-actin, JMJD2A and STMN1 (Fig. 2F-H).

### Role of JMJD2A expression and cytoskeletal remodeling in Docetaxel-Resistant CRPC cells

**Fig. 3:**
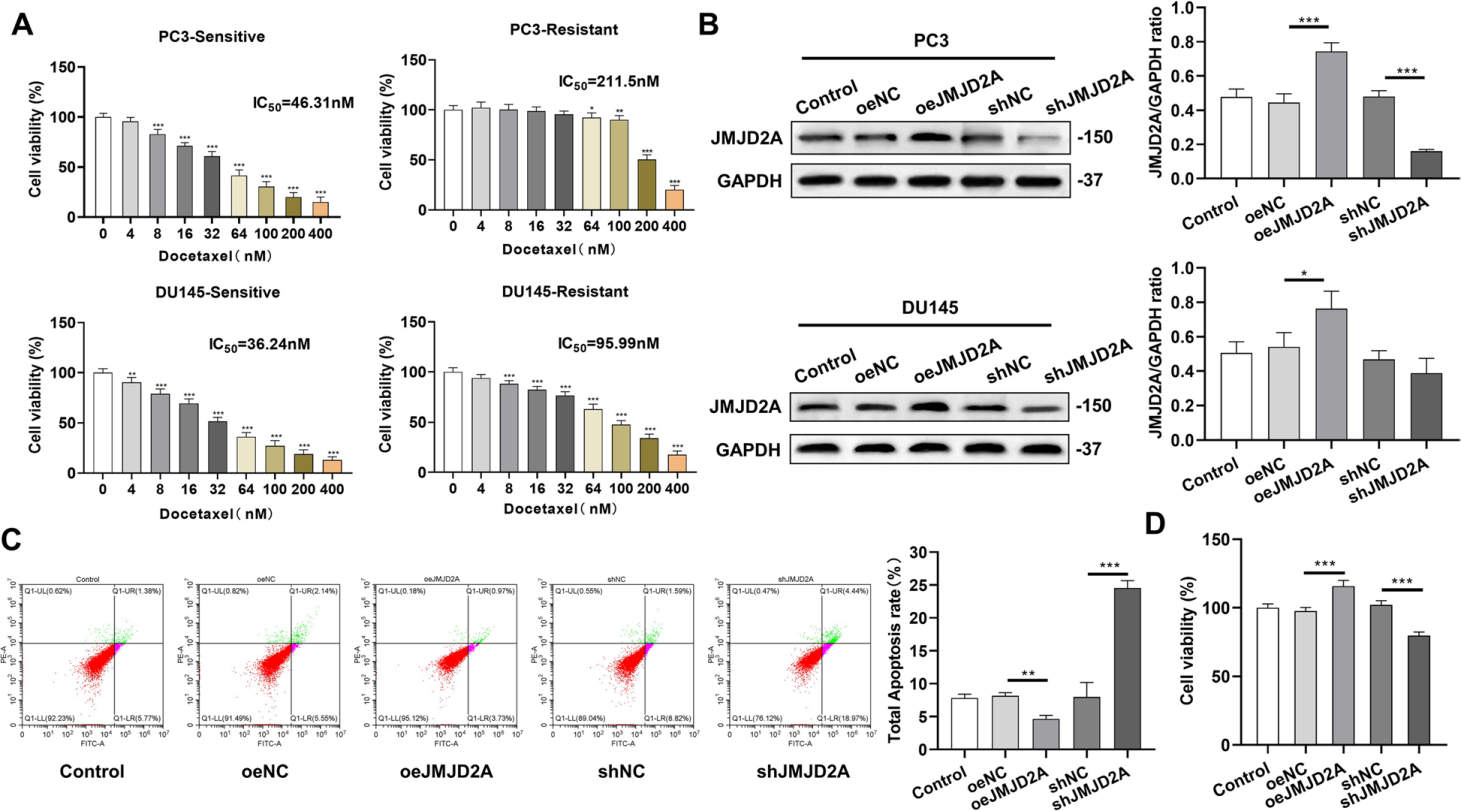
In vitro experiments exploring the effect of abnormal JMJD2A expression in prostate cancer cells. **A** Cell activity assay using sensitive and resistant strains of the human prostate cancer cell lines PC3 and DU145; *P < 0.05, **P < 0.01, ***P < 0.001, compared with 0 nM of docetaxel. **B** JMJD2A overexpression or knockdown plasmids were constructed and transfected into the prostate cancer cells, the efficiency of the transfection detected by western blot; **C** Apoptosis levels detected by flow cytometry; **D** Detection of cell proliferation by CCK-8. *P < 0.05, **P < 0.01, ***P < 0.001

The human prostate cancer cell lines PC3 and DU145 were selected and cultured for in vitro experiments. Docetaxel-resistant cell lines were constructed, and the corresponding parental cell lines were used as a control. CCK-8 results showed that the sensitivity of drug-resistant cell lines to docetaxel was significantly reduced, indicating successful construction of the drug-resistant cell lines (Fig. 3A). Next, JMJD2A overexpression or knockdown plasmids were constructed and transfected into the prostate cancer cells, and western blot was performed to confirm the efficiency of the transfection (Fig. 3B). Since better transfection effects were observed with PC3 cells, they were used in the subsequent experiments.

The cells were then divided into the following groups: Control, oeNC, oeJMJD2A, shNC and shJMJD2A. The results showed that the apoptosis of PC3 cells was significantly reduced (*P* < 0.01) and cell viability was significantly increased (*P* < 0.001) after overexpressing JMJD2A, while the opposite results were obtained after knocking down JMJD2A (Fig. 3C-D).

### Mechanism of JMJD2A regulating Cytoskeletal Remodeling to promote Docetaxel Resistance in CRPC cells

**Fig. 4:**
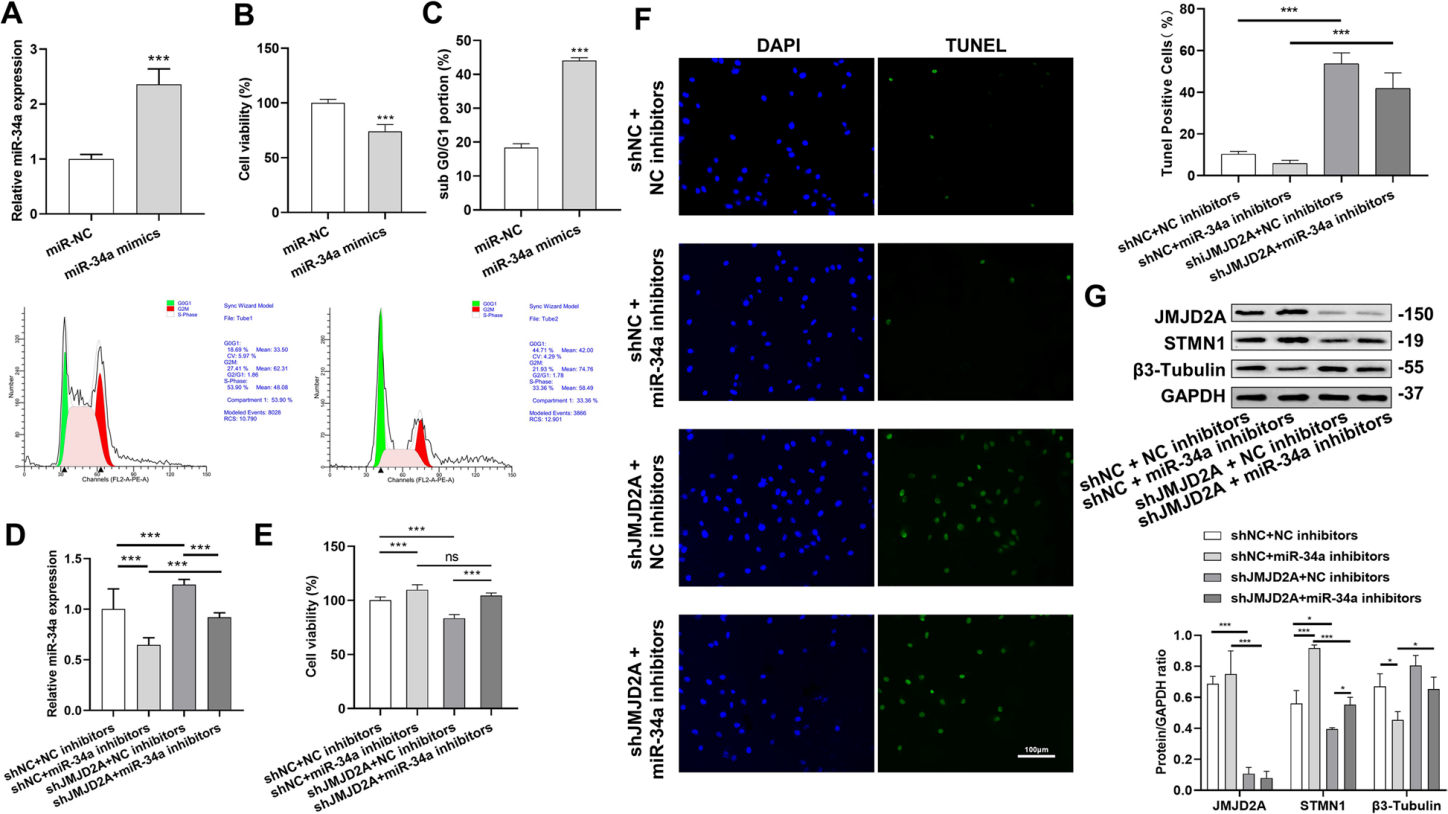
The relationship between JMJD2A and miR-34a in prostate cancer cells. **A** miR-34a overexpression detected by PCR; **B** Detection of cell proliferation affected by miRNA-34 overexpression by CCK-8; **C** Cell cycle distribution assessed by flow cytometry; **D** miR-34a expression detected by PCR; **E** Detection of cell proliferation by CCK-8; **F** TUNEL staining for assessing apoptosis; **G** The protein expression of JMJD2A, STMN1 and β3-Tubulin detected by western blot. *P < 0.05, **P < 0.01, ***P < 0.001

To investigate whether JMJD2A could regulate cytoskeletal remodeling through the miR-34a/STMN1/β3-Tubulin axis, PC3-docetaxel-resistant cell lines were cultured and constructed. JMJD2A, miR-34a, STMN1 and β3-Tubulin plasmids were transfected for single or combined intervention. Firstly, miR-34a was successfully overexpressed in vitro, and the CCK-8 results revealed that the cell proliferation in the miR-34a mimics group was significantly lower than that in the miR-34a NC group (Fig. 4A-B). Moreover, the cell cycle distribution was assessed by flow cytometry, and the results showed that miRNA-34a overexpression promoted an accumulation of cells in the G1 phase of the cell cycle (Fig. 4C), indicating that miR-34a can inhibit the growth of cancer cells and block the cell cycle in the G0/G1 phase, thereby exhibiting excellent anticancer ability. Additionally, in order to further study the mechanism of JMJD2A regulating cytoskeleton removing to promote docetaxel resistance in CRPC cells, the follow-up study was conducted. As shown in Fig. 4D, the knockdown of JMJD2A promoted the expression of miR-34a. CCK-8 and TUNEL results showed that cancer cell proliferation was significantly reduced, and apoptosis was increased in the shJMJD2A + NC inhibitors group compared with the shNC + NC inhibitors group. Meanwhile, compared with the shJMJD2A + NC inhibitors group, the cancer cell proliferation was significantly increased while apoptosis was decreased in the shJMJD2A + miR-34a inhibitors group, suggesting that the miR-34a inhibitor could restore the decreased cell proliferation and increased apoptosis caused by increased miR-24a expression induced by shJMJD2A (Fig. 4E-F). In addition, we found that after the knockdown of JMJD2A, the protein expression of STMN1 was significantly decreased; conversely, the protein expression of β3-Tubulin was significantly increased. Moreover, the addition of the miR-34a inhibitor reversed the expression trends of STMN1 and β3-Tubulin to some extent (Fig. 4G).

**Fig. 5:**
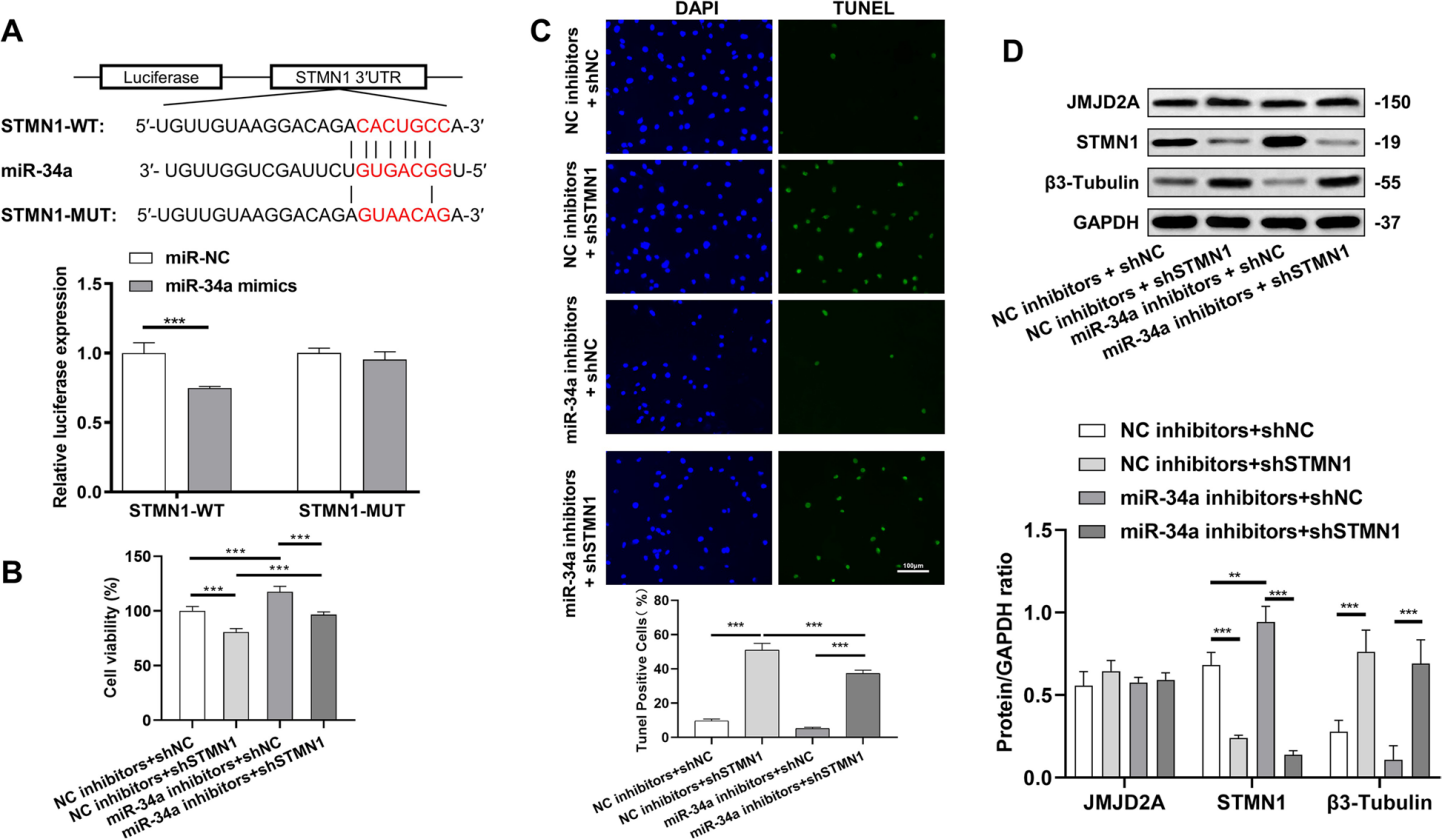
Association between miR-34a and STMN1 in prostate cancer cells. **A** Dual-luciferase assay to determine the targeting relationship between miR-34a and STMN1; **B** Detection of cell proliferation by CCK-8; **C** The protein expression of JMJD2A, STMN1 and β3-Tubulin detected by western blot; **D** TUNEL staining for assessing apoptosis. *P < 0.05, **P < 0.01, ***P < 0.001

Consistent with a previous study (23), our dual-luciferase assay results confirmed that STMN1 was a downstream target gene of miR-34a (Fig. 5A). Cell viability assay showed that the knockdown of STMN1 reduced the activity of cancer cells, and TUNEL staining showed a significant increase in apoptosis. The above changes were reversed after adding the miR-34a inhibitor (Fig. 5B-C). Moreover, western blot results showed no statistical difference in the expression of JMJD2A among the four groups. Further, adding the miR-34a inhibitor targeted and promoted the protein expression of STMN1 and inhibited the protein expression of β3-Tubulin. Notably, the protein expression of β3-Tubulin was significantly increased after knocking down STMN1 (Fig. 5D), suggesting a possible interaction between STMN1 and β3-Tubulin.

**Fig. 6:**
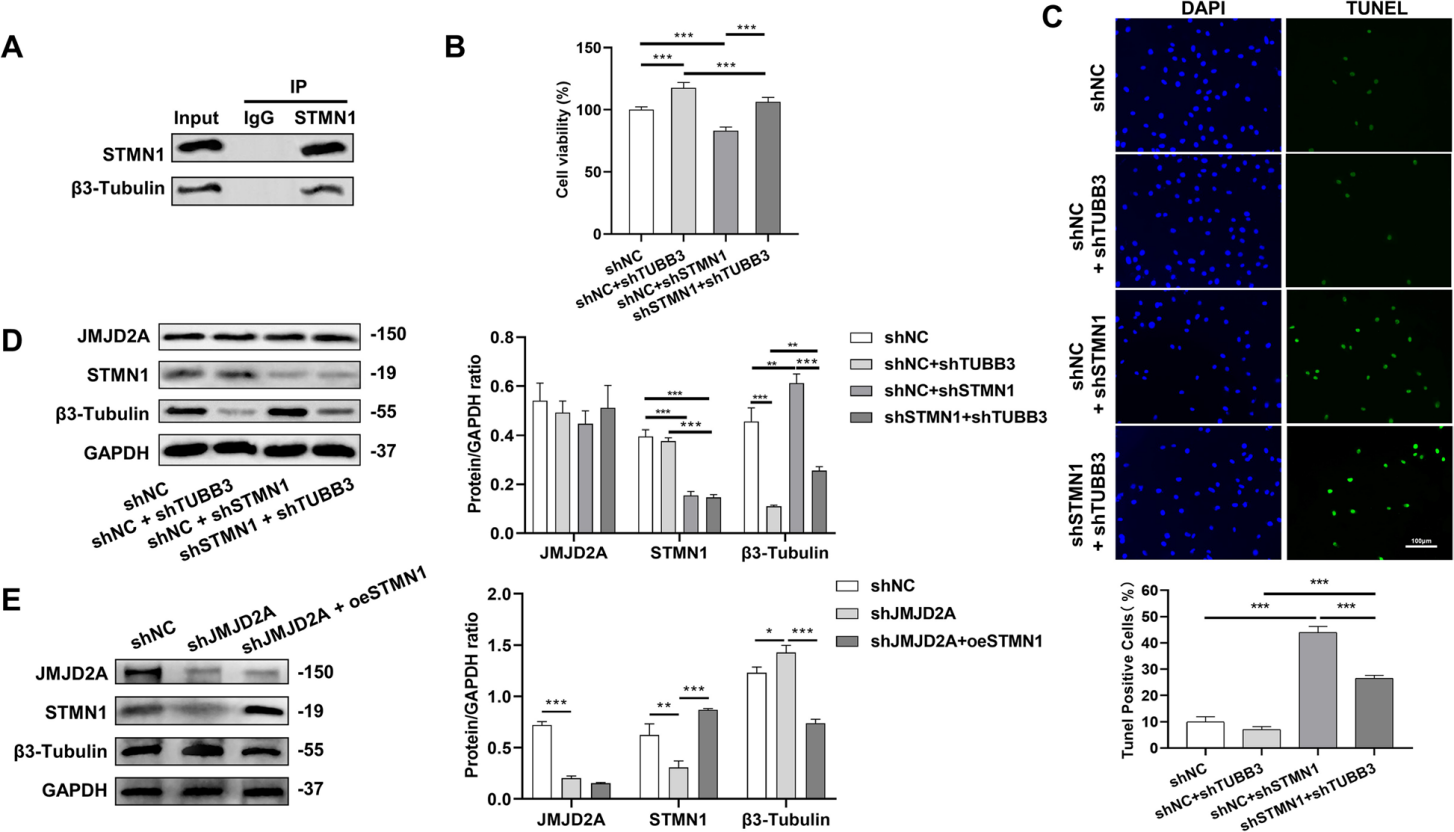
Relationship between STMN1 and β3-Tubulin in prostate cancer cells. **A** Immunoprecipitation to detect the interaction between STMN1 and β3-Tubulin; **B** Detection of cell proliferation by CCK-8; **C** TUNEL staining for assessing apoptosis; **D**-**E** The protein expression of JMJD2A, STMN1 and β3-Tubulin detected by western blot. *P < 0.05, **P < 0.01, ***P < 0.001

As shown in Fig. 6A, the co-immunoprecipitation assay showed an interaction between STMN1 and β3-Tubulin. To explore the underlying mechanism of STMN1 and β3-Tubulin in docetaxel-resistant PC3 cells, the expression of β3-Tubulin was inhibited by knocking down TUBB3 because TUBB3 encodes β3-Tubulin. Our results showed that compared with the shNC group, after knocking down TUBB3, the activity of cancer cells was significantly enhanced, while their apoptosis was significantly reduced in the shNC + shTUBB3 group. Conversely, after knocking down STMN1, compared with the shNC group, the activity of cancer cells in the shSTMN1 + shNC group was significantly decreased, while the apoptosis of cancer cells was significantly increased (Fig. 6B-C). Western blot results showed that inhibition of STMN1 expression could promote the expression of β3-Tubulin, which inhibited cancer cell proliferation and promoted cancer cell apoptosis (Fig. 6D). The experimental results to further examine whether the increase in β3-Tubulin abundance is related to JMJD2A knockdown showed that the protein expression of STMN1 in the shJMJD2A group decreased with JMJD2A knockdown, while β3-Tubulin increased. The increased expression of β3-Tubulin was reversed after STMN1 overexpression in shJMJD2A cells (Fig. 6E).

### Effects of intervening JMJD2A expression on Docetaxel-Resistant CRPC in vivo

**Fig. 7:**
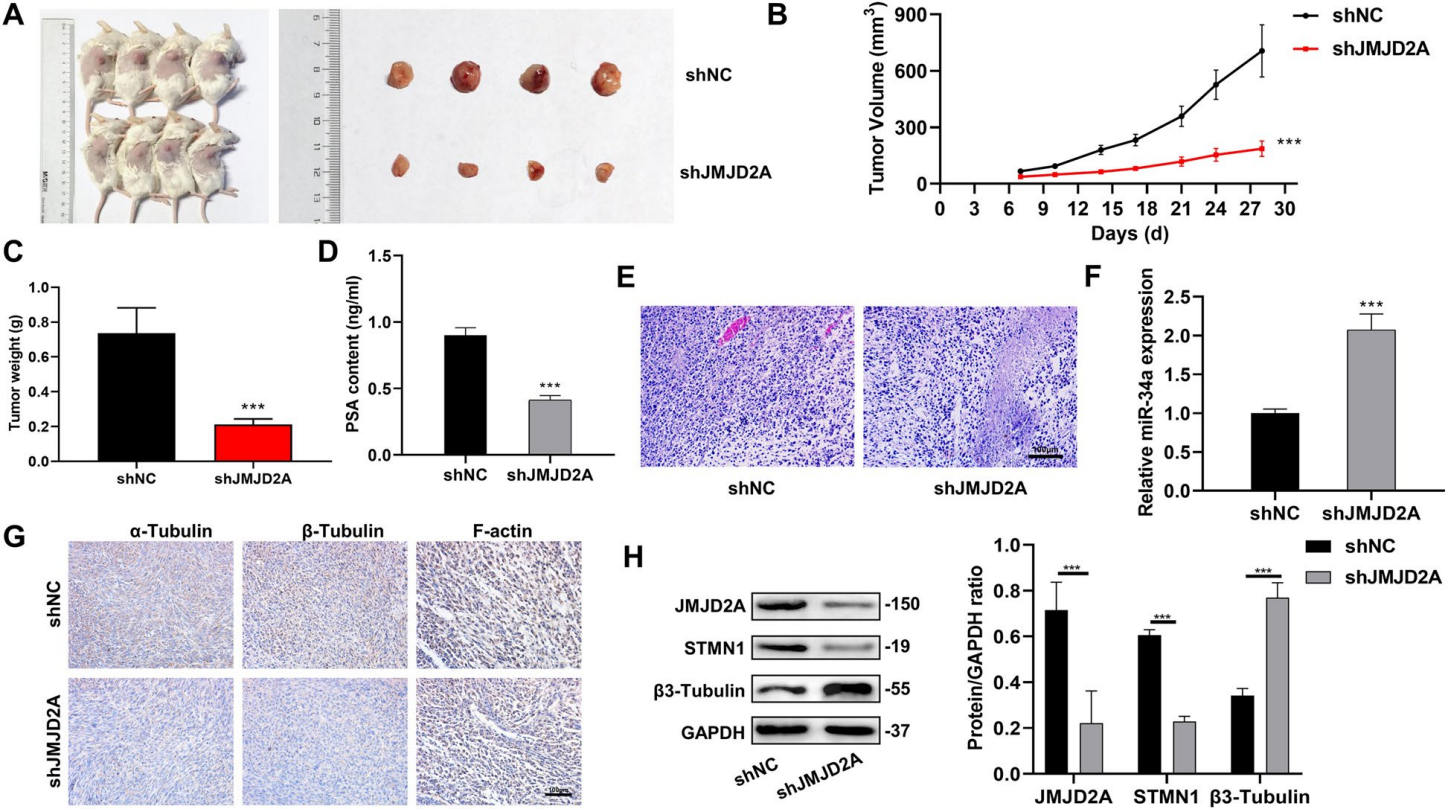
Detection of JMJD2A and cytoskeleton remodeling-related factors in CRPC docetaxel-resistant mice in in vivo experiments. **A** Mice PDX models of shNC and shJMJD2A groups; **B** Change in the tumor volume of the PDX models after treatment; **C** Change in the tumor weight of the PDX models after treatment; **D** PSA levels in mouse serum detected by ELISA; **E** The pathological changes in tissue samples observed by H&E staining; **F** miR-34a expression detected by PCR; **G** The levels of microtubulin α-Tubulin and β-Tubulin, F-actin in tissue samples observed by immunohistochemistry; H: JMJD2A, STMN1 and β3-Tubulin protein expression detected by western blot. ***P < 0.001, compared with the shNC group

To further verify the effect of JMJD2A on docetaxel-resistant CRPC, PDX animal models were constructed, and the mice were divided into an shNC (n = 4) and shJMJD2A (n = 4) group. Compared with the shNC group, the tumor volume and weight of the shJMJD2A were significantly reduced after knocking down JMJD2A (Fig. 7A-C). ELISA experiments showed that the PSA values of the shJMJD2A group were comparatively lower; concordant with pathological findings (Fig. 7D-E). In addition, the knockdown of JMJD2A resulted in significantly higher miR-34a and β3-Tubulin expression and lower STMN1 expression compared with the shNC group; consistent with the previously validated effects of the miR-34a/STMN1/β3-Tubulin axis on prostate cancer cells. Meanwhile, compared with the shNC group, the expression of the cytoskeleton-related proteins α-Tubulin, β-Tubulin and F-actin were decreased in the shJMJD2A group (Fig. 7F-H).

## Discussion

This study investigated the causes affecting docetaxel resistance through clinical studies and in vivo and in vitro models. Our findings showed that JMJD2A promoted docetaxel resistance in prostate cancer cells by regulating cytoskeleton remodeling through the miR-34a/STMN1/β3-Tubulin axis. In vivo PDX models further confirmed that the knockdown of JMJD2A could improve CRPC docetaxel-resistant. Our results strongly indicate the important functions of JMJD2A in the docetaxel-resistant CRPC.

JMJD2A is associated with tumorigenesis and is highly expressed in human cancers that have been shown to influence tumor chemotherapy sensitivity [25]. Nakagawa et al. reported that down-regulating JMJD2A significantly affected the susceptibility of gastric cancer cells to anti-cancer drugs [10]. In this study, our assessment of clinical samples from CRPC patients and in vivo experiments revealed that JMJD2A was highly expressed in the CRPC docetaxel-resistant group, whereas miR-34a expression was significantly reduced. Further, cytoskeleton-related proteins and PSA content was found to be significantly elevated in the resistant group. It has been reported that promoting the interaction of AR with the PSA promoter by activating the JMJD2A/AR signaling pathway could lead to reduced apoptosis in prostate cancer cells [25]. Thus, the involvement of JMJD2A in tumor resistance and cytoskeleton remodeling of CRPC was put forward. However, its role in anti-cancer drug resistance in CRPC therapy was yet to be further demonstrated.

The overexpression of JMJD2A in PC3 cells resulted in significant cell growth inhibition and increased apoptosis, while its knockdown yielded opposing results; suggesting JMJD2A as a promising target for treating docetaxel-resistant CRPC. Given the role of JMJD2A in gene transcription, it was worth investigating some novel factors in regulating drug susceptibility. Our results showed that miR-34a, reported to regulate apoptosis in gastric cancer cells, was negatively correlated with the expression of JMJD2A, indicating that the knockdown of JMJD2A could significantly upregulate the expression of miR-34a [26]. Additionally, JMJD2A was shown to regulate the promoter activity of miR-34a [8]. It was previously reported that miR-34a could simultaneously target multiple genes associated with apoptosis in CRPC cells without significant side effects [25]. Therefore, we hypothesized that the knockdown of JMJD2A in prostate cancer would affect the expression of miR-34a, affecting the growth and apoptosis of prostate cancer cells. Our results showed that the knockdown of JMJD2A increased the expression of miR-34a, significantly decreased cell viability and significantly increased apoptosis; confirming our hypothesis. Wang et al. showed that miR-34a inhibited the growth of CRPC xenografts by suppressing the proliferation of CRPC cells PC-3 and promoting apoptosis [27]. Similarly, our in vivo results showed that miR-34a was significantly elevated in the shJMJD2A group and was associated with significant tumor growth inhibition. However, the mechanism of JMJD2A and miR-34a in docetaxel-resistant CRPC therapy remained largely unknown.

Studies have found that the miR-34a/STMN1/β3-Tubulin axis had a role in regulating the microtubule network of tumor cells, miR-34a could directly inhibit the expression of STMN1, and upregulating β3-Tubulin led to the destruction of microtubule networks and cell death [28], providing a theoretical basis for our results. Our study showed that inhibition of JMJD2A increased the expression of miR-34a and β3-Tubulin while decreasing STMN1 expression. Further, dual-luciferase assays demonstrated that miR-34a could target STMN1, a ubiquitous cytoplasmic protein that regulates microtubule dynamics, consistent with previous reports [20]. We found that inhibiting miR-34a expression promoted STMN1 and inhibited β3-Tubulin, which decreased cancer cell death and increased apoptosis. Furthermore, co-immunoprecipitation assays revealed an interaction between STMN1 and β3-Tubulin and that inhibition of STMN1 expression could promote the expression of β3-Tubulin, thereby inhibiting cancer cell proliferation and promoting cancer cell apoptosis. The above experiments results validated that the miR-34a/STMN1/β3-Tubulin axis could be the pathway regulating the microtubule network of tumor cells in prostate cancer. Moreover, PDX animal models were constructed to further verify the effects of JMJD2A on docetaxel-resistant CRPC, and the results showed that inhibiting JMJD2A expression in CRPC docetaxel-resistant mice could regulate the miR-34a/STMN1/β3-Tubulin axis, thereby inhibiting cytoskeletal proliferation in CRPC docetaxel-resistant mice.

## Supplementary Information

**Additional file 1.**

## Data Availability

Data will be made available from corresponding author upon reasonable request.

## Abbreviations

CRPC: Castration-resistant prostate cancer
ADT: Androgen deprivation therapy
JMJD2A: Jumonji domain 2A
STMN1: Stathmin 1
SPF: Specific pathogen-free
FBS: Fetal bovine serum
PBS: Phosphate-buffered saline
TBST: Tris-buffered saline
PI: Propidium iodide
CCK-8: Cell Counting Kit-8
ELISA: Enzyme-linked immunosorbent assay
PDX: Patient-derived xenograft
Co-IP: Co-immunoprecipitation
WT: Wild-type
MT: Mutant
IHC: Immunohistochemical
