# Supplementary material for: JMJD2A participates in cytoskeletal remodeling to regulate castration-resistant prostate cancer docetaxel resistance
Source: BMC Cancer. 2023 May 10;23:423. doi: 10.1186/s12885-023-10915-1 (PMC10170801; doi:10.1186/s12885-023-10915-1)
Supplement: Supplementary file 1 — Additional file 1. [file 12885_2023_10915_MOESM1_ESM.docx]

**Figure 1D:**

**Original image: JMJD2A**

**Original image: STMN1**

**Original image: β3-Tubulin**

**Original image: GAPDH**

**Figure 2H:**

**Original image: JMJD2A**

**Original image: STMN1**

**Original image: β3-Tubulin**

**Original image: GAPDH**

**Figure 3B:**

**Original image: JMJD2A (PC3)**

**Original image: GAPDH (PC3)**

**Original image: JMJD2A (DU145)**

**Original image: GAPDH (DU145)**

**Figure 4G:**

**Original image: JMJD2A**

**Original image: STMN1**

**Original image: β3-Tubulin**

**Original image: GAPDH**

**Figure 5D:**

**Original image: JMJD2A**

**Original image: STMN1**

**Original image: β3-Tubulin**

**Original image: GAPDH**

**Figure 6A:**

**Original image:** **STMN1**

**Original image:** **β3-Tubulin**

**Figure 6D:**

**Original image: JMJD2A**

**Original image: STMN1**

**Original image: β3-Tubulin**

**Original image: GAPDH**

**Figure 6E:**

**Original image: JMJD2A**

**Original image: STMN1**

**Original image: β3-Tubulin**

**Original image: GAPDH**

**Figure 7H:**

**Original image: JMJD2A**

**Original image: STMN1**

**Original image: β3-Tubulin**

**Original image: GAPDH**
